# Factors That Influence the Prescription of Antibiotic Therapy at the End-of-Life: Construction and Validation of a Scale

**DOI:** 10.7759/cureus.31689

**Published:** 2022-11-20

**Authors:** Elsa Campoa, Paulo Reis-Pina

**Affiliations:** 1 Medical Oncology, University Hospital Center of Algarve, Faro, PRT; 2 Internal Medicine, Casa de Saúde da Idanha, Lisbon, PRT

**Keywords:** palliative treatment, prescription drugs, scale composition, supportive and palliative care, antibacterial therapy

## Abstract

Introduction: End-of-life care is frequently discussed in clinical practice. Non-beneficial treatments and the need for decision-making regarding therapeutic institutions are increasingly addressed. There are no guidelines regarding prescribing or de-prescribing antibiotic therapy at the end of life, which depends on clinical decisions. In this study, we developed a scale to assess the factors influencing clinicians' decisions when prescribing antimicrobial agents.

Methods: This is a quantitative, exploratory, and descriptive study. After the literature review, the scale was constructed with an analysis of internal consistency and temporal stability. It was applied online together with a sociodemographic and clinical questionnaire. Statistical analysis of the scale, its construction, and final validation were performed.

Results: A total of 196 physicians participated in this study (76.5% female, 78.6% aged <40 years), 60.2% specialists, and 35.7% without palliative care training. Almost all of the participants (89.9%) reported having end-of-life care concerns with a high frequency. In this study, a scale was developed to assess factors associated with the prescription of antibiotic therapy in end-of-life patients. This scale revealed the presence of 3 factors: infection, patient/illness, and symptoms. Together, the three factors explain 57.4% of the clinician's decisions. The factors associated with symptoms were the most predominant in decision-making compared to those associated with infection.

Conclusions: Among the multiple factors that may influence the institution of antibiotic therapy at the end of life, symptomatic control is the most important factor.

## Introduction

The field of Palliative Care (PC) has shown increasing interest and development among physicians, with a focus on promoting the best care and improving the quality of life (QoL) of patients with chronic, progressive, and irreversible diseases, whether oncologic or not. In this sense, ethical and clinical questions regarding procedures and measures to be instituted in the care plan are constantly being raised. Data from a meta-analysis [1] showed rates of up to 38% in the use of unnecessary intervention measures. Thus, resuscitation measures, invasive ventilation, chemotherapy, and other treatments are increasingly being questioned. Physicians even less address the institution of antibiotic therapy (ABT) in specialized PC teams.

Thus, the health professional must have the ability and discernment to distinguish the patient's needs from futile therapies unsuitable to the patient's care plan or life expectancy, with poor/no clinical benefit. This process involves a careful, multidisciplinary assessment of the patient and his/her family [1-3]. Therapies are recommended whenever they promote symptomatic relief, with a balanced balance between risk and benefit [4].

End-of-life (EoL) prescription of ABT occurs with high frequency, with data in the literature reaching approximately 90% in the last week of the life of cancer patients in hospitalization [5-8] and 42% in the last two weeks of life of patients with advanced dementia in nursing homes or other institutions [9].

Despite several studies seeking to assess the use of ABT in EoL, most of the factors that motivate their use are unclear, with most studies referring to the clinician's decision/judgment or in the presence of symptoms of infection or fever/changes in inflammatory parameters [10-12]. It is then described that patients with poor performance status, advanced disease stage, and lack of clinical benefit will be good indicators for antimicrobial therapy not to be initiated [5,13].

Several factors to be considered are described in the literature, but it is not established which are the most important in decision-making. Based on this assumption, this study was developed to understand which of the factors described in the literature are most important for Portuguese physicians when deciding on the institution of ABT.

## Materials and methods

This is a quantitative, exploratory, descriptive, cross-sectional, observational, correlational/inferential study [14]. After a literature review and peer discussion, the final assessment instrument was created. A pilot application was conducted for linguistic correction and internal evaluation of the instrument, which, after adequate internal consistency and retest values, was used to develop an online self-completion questionnaire.

The final questionnaire, in the Portuguese language, was applied between September and December 2021. Inclusion criteria were: informed consent; physicians in clinical practice in Portugal; physicians dealing with EoL situations, with or without previous training in PC. Patients who did not meet the inclusion criteria would be excluded.

The collection procedure was carried out by sharing a link to access the online questionnaire through peer sharing and disclosure by the following societies Portuguese Society of Internal Medicine, Portuguese Society of Oncology, Portuguese Association of Palliative Care, and Portuguese Association of General and Family Medicine.

The Ethics Committee approved this study of the Lisbon Academic Medical Center.

## Results

Characterization of the sample

Of the total 196 participants, 76.5% were female (n=150). The most frequent age group was 30-40 years in 60.7% of cases. With an excellent geographical distribution of the participants, the greatest participation of physicians from the Lisbon region (33.2%) and the Algarve (29.1%) should be noted.

Most were either residents (39.8%) or specialists with up to 5 years of completion of their specialty (30.1%), without PC training (35.7%), or with short-term training (25.0%). In 17.9% (n=35) of the cases, it was verified that the physicians collaborated in PC teams; 80% belonged to intra-hospital teams and 20% to community teams. The global characterization of the participants is shown in table 1.

**Table 1:** Global characterization of the participants (n=196) Demographic characterization of the participants, and their professional characteristics (area, length of experience, PC training).

	n	%
Sex		
Male	46	23.5
Female	150	76.5
Age		
<30 years old	35	17.9
30-40 years old	119	60.7
40-50 years	28	14.3
>50 years	14	7.1
Geographic Area		
North	38	19.4
Center	24	12.2
Lisbon	65	33.2
Alentejo	5	2.6
Algarve	57	29.1
Madeira/Azores	7	3.6
Specialty Area		
General Surgery	8	4.1
Internal Medicine	75	38.3
General and Family Medicine	37	18.9
Medical Oncology	37	18.9
Other Specialties	39	19.9
Specialty Years		
Resident 1-3 years	30	15.3
Resident 3-6 years	48	24.5
Specialist <1-5 years	59	30.1
Specialist 5-10 years	32	16.3
Specialist >10 years	27	13.8
Palliative Care Training		
None	70	35.7
Course/internship <= 30 hours	49	25.0
Course/internship > 30 hours	33	16.8
Post-graduation	29	14.8
Master's degree	13	6.6
PhD	2	1.0
Collaboration in Palliative Care Team		
No	161	82.1
Yes	35	17.9
Community Care Team	7	20.0
Intra-hospital team	28	80.0
Work Place		
University Hospital Center	77	39.3
Central Hospital	26	13.3
Peripheral Hospital	48	24.5
Health Center generalistic unit	12	6.1
Health center special Unit	20	10.2
Hospital/Private Institution	13	6.6

Initial data collection instrument

After reviewing the existing literature, we prepared a questionnaire with a Likert scale from 1 to 6, where 1 corresponds to disagree strongly and 6 to strongly agree. After the literature review, predominantly in existing retrospective studies, we searched for variables/factors interpreted as characteristics of patients who started antibiotic therapy. With this information, statements were produced to evaluate the clinicians' positioning in selecting antibiotic therapy according to the various cases. According to the literature, there were clinical variables related to the patient and his/her general condition or associated with biochemical parameters and infection symptoms. The scale aimed to assess the importance health professionals attach to each factor when deciding to use ABT in EoL patients.

To assess the clarity and understanding of the items, a pilot test was conducted with a convenience sample of 10 physicians from the study population. Based on the results, the necessary clarifications or exclusion of confounding items were made to present the final scale (table 2).

**Table 2:** Homogeneity statistics of the items and internal consistency coefficients (Cronbach's alpha) of the initial scale applied to the pilot group.

Items		n	Correlation without item	Cronbach's alpha (excluding item)
1	Antibiotherapy should be initiated whenever there is a benefit in symptomatic control	10	0.642	0.671
2	In a patient with no capacity for family-related life, I choose not to institute antibiotherapy	10	0.321	0.702
3	The presence of fever alone is not an indicator of antibiotherapy	10	-0.194	0.737
4	In an elderly patient with cachexia that appears with fever and prostration, I should initiate antibiotherapy	10	-0.588	0.776
5	In the presence of leukocytosis and increased C-reactive protein, I should initiate antibiotherapy	10	0.759	0.652
6	The degree of dependence of the patient on activities of daily living influences the decision to initiate antibiotherapy	10	0.467	0.682
7	Dyspnea and increased secretions at the end of life are better treated by other drugs than antibiotherapy	10	0.247	0.706
8	A patient with a fever should be started on antibiotherapy because he may have an infection	10	0.236	0.708
9	It should be started whenever there is a suspicion of infection.	10	0.641	0.672
10	Age is an important factor in the decision to start antibiotherapy	10	0.556	0.671
11	A patient with increased inflammatory parameters always benefits from antibiotherapy	10	0.447	0.698
12	Metastatic cancer disease unresponsive to directed treatment is a major factor in the decision not to institute antibiotherapy	10	0.073	0.742
13	Increased C-reactive protein may not be associated with the presence of infection and therefore, antibiotherapy should not be initiated	10	0.314	0.702
14	In a patient with end-organ failure with suspected infection, antibiotherapy should be instituted	10	0.318	0.700
15	A patient with increased secretions and dyspnea benefits from antibiotherapy for symptomatic control	10	0.265	0.705
16	If its institution brings no benefit to the general condition of the patient, it should not be performed	10	0.321	0.704
17	In a patient with symptoms and leukocytosis, I should start empirical antibiotherapy	10	0.615	0.670
18	In a patient with renal failure in whom hemodialysis has been suspended, there is a benefit in treating an infection with antibiotherapy	10	0.199	0.712
19	Fever and leukocytosis are signs of infection and antibiotherapy should be started	10	0.379	0.696
20	In a patient with end-stage lung cancer should I treat a respiratory infection with antibiotherapy	10	0.079	0.718
Total Cronbach's alpha		0.745		

The final scale contained a set of randomly presented items. The items were presented in both positive and inverse forms. Items 3, 4, 7, 13, 14, 18, and 20 were reverse-rated.

This scale sought to identify 2 factors: factors associated with the patient and disease; and associated with infection (clinical and inflammatory mediators). The items corresponding to factors associated with the patient and disease: 1, 2, 4, 6, 10, 12, 14, 16, 18, and 20. The items corresponding to infection-associated factors: 3, 5, 7, 8, 9, 11, 13, 15, 17, and 19. Higher scores in each group are related to the most prevalent factors in the clinician's decision-making at the moment of ABT institution.

Cronbach's alpha coefficient assessed the internal consistency of the scale. Test-retest methodology evaluated Temporal stability by reapplying the test to a convenience sample of 10 participants 15 days after the first application.

Construction and validation of the assessment instrument

The total scale was initially submitted to a validation of the understanding of the items by two physicians (one from Medical Oncology and one from Internal Medicine), which resulted in a Cronbach's alpha of 0.779 and an interrater correlation of 64.4%.

After this application, and after adjustments were made to the sentence construction to make them more understandable, the questionnaire was applied to a pilot group of 10 physicians in a convenience sample, residents (60%), and specialists (40%) in Medical Oncology and Internal Medicine (5 participants each). In this pilot evaluation, we analyzed the scale's internal consistency and applied a retest with a 15-day time interval between the two applications.

The pilot group had a Cronbach's alpha of 0.745. In the analysis for each item, as presented in table 2, it was found that only with the exclusion of item 4 could there be a slight difference in Cronbach's alpha, which was limited, so we chose to keep all original items. The interclass Correlation (test-retest) was 0.821. The interrater consistency values in this pilot group were between 41.6% and 87.9%.

In the analysis with the final total population (n=196), there was a decrease in Cronbach's alpha to values of 0.315. To understand the factors contributing to this result, we performed item correlation and Cronbach's alpha analysis with item deletion.

One item was eliminated at a time, seeking to verify Cronbach's alpha at the time of its elimination while maintaining a model explanatory capacity > 50% in the factor analysis. Thus, the final scale, composed of 13 items, had a Cronbach's alpha of 0.729, whose characteristics are shown in table 3. Factor analysis was performed to infer decisive factors associated with infectious changes (clinical or inflammatory) or associated with the patient and disease.

**Table 3:** Statistics of homogeneity of the items and internal consistency coefficients (Cronbach's alpha) of the final scale - total population

Items		n	Correlation without item	Cronbach's alpha (excluding item)
1	Antibiotherapy should be initiated whenever there is a benefit in symptomatic control	196	0.333	0.714
2	In a patient with no capacity for family-related life, I choose not to institute ATB	196	0.123	0.741
5	In the presence of leukocytosis and increased C-reactive protein, I should initiate antibiotherapy	196	0.542	0.688
6	The degree of dependence of the patient on activities of daily living influences the decision to initiate antibiotherapy	196	0.289	0.723
8	In a patient with a fever, I should initiate ATB because he may have an infection	196	0.493	0.702
9	It should be started whenever there is a suspicion of infection.	196	0.394	0.707
10	Age is an important factor in the decision to start antibiotherapy	196	0.354	0.712
11	A patient with increased inflammatory parameters always benefits from antibiotherapy	196	0.413	0.710
12	Metastatic cancer disease with no response to directed treatment is a major factor in the decision not to institute antibiotherapy	196	0.272	0.725
15	A patient with increased secretions and dyspnea benefits from antibiotherapy for symptomatic control	196	0.276	0.721
16	If its institution does not benefit the general condition of the patient, it should not be performed	196	0.308	0.717
17	Should I initiate empirical antibiotherapy in a patient with symptoms and leukocytosis	196	0.462	0.697
19	Fever and leukocytosis are signs of infection and antibiotherapy should be initiated	196	0.471	0.699
Total Cronbach's alpha		0.729		

The Kaiser-Keyer-Olkin (KMO) test and Bartlett's index were used to test the correlation quality between variables and the factor matrix's validity. The significance level of the tests was set at 0.05. According to Hill et al. (2012) [15], obtaining a Bartlett index of 0.000 and a KMO equal to 0.788 allowed for validating the occurrence of average correlations between the variables.

A factor analysis of the principal components was performed, with orthogonal varimax rotation, to form categories. The factorial solution allowed the selection of 3 factors with latent roots greater than 1, which together explained 57.42% of the total variance (Table 4).

**Table 4:** Factor analysis of the scale by the Principal Components Analysis method with Varimax rotation

Items		Factor 1	Factor 2	Factor 3
1	Antibiotherapy should be initiated whenever there is a benefit in symptomatic control	0.236	-0.072	0.814
2	In a patient with no capacity for family-related life, I choose not to institute antibiotherapy	-0.196	0.679	0.119
5	In the presence of leukocytosis and increased C-reactive protein, I should initiate antibiotherapy	0.722	0.141	0.140
6	The degree of dependence of the patient on activities of daily living influences the decision to initiate antibiotherapy	-0.026	0.807	0.115
8	In a patient with a fever, I should initiate antibiotherapy because he may have an infection	0.744	0.079	0.011
9	It should be started whenever there is a suspicion of infection.	0.682	-0.151	0.214
10	Age is an important factor in the decision to start antibiotherapy	0.267	0.685	-0.152
11	A patient with increased inflammatory parameters always benefits from antibiotherapy	0.774	0.060	-0.212
12	Metastatic cancer disease with no response to directed treatment is a major factor in the decision not to institute antibiotherapy	0.040	0.746	0.028
15	A patient with increased secretions and dyspnea benefits from antibiotherapy for symptomatic control	0.418	0.009	0.148
16	If its institution does not benefit the general condition of the patient, it should not be performed	0.002	0.251	0.783
17	Should I initiate empirical antibiotherapy in a patient with symptoms and leukocytosis	0.623	-0.100	0.456
19	Fever and leukocytosis are signs of infection and antibiotherapy should be initiated	0.809	-0.057	0.028
Total variance explained = 57.42%.		28.46%	17.62%	11.34%
Kaiser-Meyer-Olkin Measure of Sample Adequacy		0.788		
Kaiser-Meyer-Olkin Measure of Sample Adequacy		733.05 p<0.000		

Factor 1 was designated as a factor associated with infection and explained 28.46% of the total variance, consisting of items 5, 8, 9, 11, 15, 17, and 19.

Factor 2 was designated as a factor associated with the patient/illness and explained 17.62% of the total variance, consisting of items 2, 6, 10, and 12.

Factor 3 was designated symptom-related factor and explained 11.34% of the total variance, consisting of items 1 and 16.

Overall the scale considered that higher scores are associated with the decision to use antibiotic therapy according to these factors. The higher values are associated with their greater preponderance in decision-making. When objectifying factorial results to compare the weighting degree of each factor, the following adjustment calculation should be carried out: Factor 1 = total result factor 1 x1; Factor 2 = total result factor 2 x1.75; Factor 3 = total result factor 3 x3.5. Each factor has a minimum of 7 and a maximum of 42. The final version of the scale is shown in Table 5.

**Table 5:** Final scale of factors influencing the institution of antibiotic therapy at the end of life For each of the following statements please mark your position, considering 1 strongly disagree and 6 strongly agree.

		1 Strongly Disagree	2	3	4	5	6 Strongly Agree
1	Antibiotherapy should be initiated whenever there is a benefit in symptomatic control						
2	In a patient with no capacity for family-related life, I choose not to institute antibiotherapy						
3	In the presence of leukocytosis and increased C-reactive protein, I should initiate						
4	The degree of dependence of the patient on activities of daily living influences the decision to initiate antibiotherapy						
5	In a patient with a fever, I should initiate antibiotherapy because he may have an infection						
6	It should be started whenever there is a suspicion of infection.						
7	Age is an important factor in the decision to start antibiotherapy						
8	A patient with increased inflammatory parameters always benefits from antibiotherapy						
9	Metastatic cancer disease with no response to directed treatment is a major factor in the decision not to institute antibiotherapy						
10	A patient with increased secretions and dyspnea benefits from antibiotherapy for symptomatic control						
11	If the institution of antibiotherapy does not benefit the general condition of the patient, it should not be performed						
12	If the patient has symptoms and leukocytosis, I should start empirical antibiotherapy						
13	Fever and leukocytosis are signs of infection and antibiotherapy should be initiated.						

Overall results of the scale of assessment of factors influencing the prescription of antibiotic therapy in end-of-life patients

In the global assessment of the final scale of 13 items, the total mean values were 38.48 (SD 8.507, minimum 18, maximum 62). In the analysis of the three factors on the scale, it was found that the highest values occurred in the factors associated with symptoms and that the lowest scale values corresponded to those associated with infection, as shown in Table 6.

**Table 6:** Descriptive Statistics and T-Test for Comparison of Means of Factors Associated with Prescribing

	Factor 1: Associated with infection	Factor 2: Associated with patient/illness	Factor 3: Associated with symptoms
Mean	16.66	20.60	35.18
Median	16.0	21.0	38.5
Standard Deviation	6.04	7.79	7.72
Minimum	7	7	11
Maximum	34	42	42
T-test	t=38.59 p<0.01	t=37.01 p<0.01	t=63.84 p<0.01

When the mean values of each factor are compared, the difference between them is statistically significant (p<0.01). These results suggest that the factors associated with symptoms are the most important in the clinician's decision to use ABT, and those associated with infection are the least important.

The correlation analysis between the 3 factors shows that physicians whom most value factors associated with infection tend to also value factors associated with symptoms, with a statistically significant correlation (r=0.294, p<0.01). No statistically significant relationships were found between the remaining factors.

## Discussion

In the scale developed in this study, 3 factors associated with the prescription of ABT were highlighted: infection, patient/illness, and symptoms. This scale showed good internal consistency (Cronbach's alpha of 0.729), as well as good validity of the Correlation between variables and in the development of factors (Bartlett index 0.000 and KMO 0.788), and overall the 3 verified factors explain 57.4% [15].

Among the 3 factors, the factor associated with symptoms was given a higher weighting than the other factors. The factor associated with infection was given a lower weighting; these differences were statistically significant (p<0.01). These data reflect the clinicians' particular concern for the symptomatology presented by patients and their care. The higher weighting assigned to symptom-related factors is consistent with the existing literature, which considers that the use of ABT should be carried out whenever it is associated with the patient's clinical benefit, considering the treatment focus on symptom benefit and control [4,8,11].

On the other hand, the factors associated with infection had the least weight in the decision-making process. It is considered that in EoL patients, even in the presence of a confirmed infection, the use of ABT is not always beneficial, as some authors have previously considered that it is only associated with prolonging the patient's suffering [16]. However, these data disagree with the frequencies in the literature regarding the use of ABT upon suspicion of infection, with several national and international series reflecting high rates of therapy introduction upon the presence of infection (suspected or confirmed), including difficulties in discontinuing it after failure in the first days [10,17-18]. The discrepancies in these results may be associated with the fact that the questionnaire represents fictitious statements that diverge from real situations, with a component of the doctor-patient relationship, and pressures made by patients or families, which may alter clinicians' decisions at certain times.

Patient/illness-related factors remained the intermediate preponderance factor. These factors, mainly associated with the patient's general condition, stage, and phase of disease evolution, may be associated with greater subjectivity in their assessment. However, their importance in decision-making is highlighted given the high frequency of EoL patients with no relationship life or potential for recovery, who are treated with ABT but do not have the best symptomatic supportive care [5].

Considering these three factors, which together explained 57.4% of the clinicians' decisions to use ABT, there is a positive correlation between factors associated with infection and those associated with symptoms. These results reflect their concern with symptomatic control since most clinicians believe that an infectious condition may be related to the lack of symptomatic control, thus their interconnection. In this sense, the factors associated with infection may influence the clinician's decision-making more due to the lack of symptomatic control they promote and not due to the infectious disease itself. However, in clinical practice, we observed higher frequencies of antibiotic prescriptions than desirable, considering the real benefit for the patient [8,16,19]. However, we found that other factors involved in the clinicians' decision are not included in the determinants of this scale, for which future studies will be needed.

Limitations and strengths

As a limitation, the difficulty in distributing/applying the questionnaire at a national level is denoted, with total participation lower than expected. All participants had a special interest in the area and were not innocuous for subgroup comparisons. Likewise, given that these were fictitious scenarios, verifying the possibility of the answers being associated with what the participants know they should do and not with the decision they would effectively make under a real scenario is necessary.

Since the scale explains 57.4% of the factors associated with antibiotic prescribing, further studies will be necessary for the future to allow us to recognize other variables not included in this instrument, probably in a study designed with another, a more qualitative methodology that allows us to explore in more depth the reasons behind the decisions in clinical practice. The development of this instrument, validated for the Portuguese population, allows for the recognition of the clinicians' main weighting factors, which may help assess training needs in these areas and, consequently, the results obtained after this training.

## Conclusions

Symptomatic control is one of the most common themes in the literature when searching for PC. However, regarding the care of EoL and measures to be instituted, there are no precise definitions, guidelines, or directives which allow common decision-making among all health professionals. This study allowed the development of a scale that explains more than 50% of the factors present at the moment of the decision to institute ABT in EoL patients by weighting factors associated: with infection, patient/illness, or symptoms. Statistically significant differences were found in the degree of predominance of each factor in the clinicians' decision-making, with the most predominant factor being that associated with symptoms. Thus, it is determined that in clinical practice, the main focus is on symptomatic control. The presumptive diagnosis of infection is the least important factor for clinicians when making decisions regarding the institution of antimicrobial therapy.
